# Using ambient AI in clinical consultations: reframing policy around clinical audit and patient safety

**DOI:** 10.3389/fdgth.2026.1918841

**Published:** 2026-08-17

**Authors:** Vincent Misrai, Alena Bruchon, Prokar Dasgupta, Shahrokh F. Shariat, Jeremy Y. C. Teoh, Jean-Michel Loubes, Sameer Pujari, Antoine Piau

**Affiliations:** 1 Department of Urology, Clinique Pasteur, Toulouse, France; 2 Public and Private Cloud Department, Airbus SAS, Toulouse, France; 3 The London Clinic, London, United Kingdom; 4 Department of Surgery, King’s College London, London, United Kingdom; 5 Department of Urology, Comprehensive Cancer Center, Medical University of Vienna, Vienna, Austria; 6 Department of Urology, Weill Cornell Medical College, New York, NY, United States; 7 Department of Urology, University of Texas Southwestern, Dallas, TX, United States; 8 S.H. Ho Urology Centre, Department of Surgery, Faculty of Medicine, The Chinese University of Hong Kong, Hong Kong, China; 9 Institut de Mathématiques de Toulouse, INSA, Université Toulouse 3, CNRS, Toulouse, France; 10 Artificial and Natural Intelligence Toulouse Institute (3IA ANITI), Toulouse, France; 11 Department of Data, Digital Health, Analytics and AI, World Health Organization, Geneva, Switzerland; 12 CERPOP, INSERM, Université Toulouse 3, Toulouse, France; 13 IHU HealthAge, University Hospital of Toulouse, Toulouse, France; 14 Medical, Ethics and Innovation Department, Clariane Group, Paris, France

**Keywords:** ambient artificial intelligence, audit and feedback, clinical audit, clinical consultation recording, data governance, health equity, health policy, patient safety

## Abstract

Recent work has mapped what is legally permitted, commercially practiced, and technically coming for ambient AI in clinical care, yet it has not addressed the prior question of what the recording is for. This Perspective offers a policy argument, not an empirical evaluation. Documentation, workflow efficiency, patient recall, medico-legal transparency, and quality improvement are all legitimate purposes of consultation recording; we propose that one further, high-value purpose has been under-recognized: clinical audit. The high-fidelity transcript makes communicated clinical reasoning observable at scale, aligning consultation recording with the WHO Global Patient Safety Action Plan 2021–2030. Examining the regulatory, vendor, patient, and clinician perspectives, we argue that European law permits this purpose but does not supply it, and that consent should be reframed around patient protection rather than institutional convenience, a protective framing that is itself a hypothesis to be tested. Vendors, in turn, must redesign their products to be audit-ready, with transcription accuracy reported transparently across languages and populations. The efficiency gains that will drive adoption and the audit purpose that could convert adoption into patient value draw on the same artifact and need not compete. As a testable policy proposal, we call for a minimum audit-ready transcript standard, piloted in parallel across high-income and low- and middle-income settings, so that the clinical value of ambient AI is built in from the start rather than retrofitted after high-income adoption.

## 1 Introduction

Recent work has begun to set out the contours of ambient artificial intelligence (AI) in clinical practice. Anderson and colleagues describe the legal exposure associated with consultation recordings (1). Gerke and Simon document, for the first time, the heterogeneity of vendor practices for retaining audio, transcripts, and clinical notes, and highlight the privacy considerations that follow (2). Earlier, Barr and colleagues urged the medical community to prepare for the widespread adoption of clinic visit recording (3).

Throughout this Perspective, “ambient AI” refers to systems, often called AI scribes, that passively capture the clinician–patient conversation and generate a transcript and draft documentation from it. These systems are distinct from real-time clinical decision support, which intervenes during the encounter, and from conventional retrospective audit tools, which work from coded data and written notes rather than from the conversation itself; our proposal concerns the retrospective use of the transcripts ambient systems already produce. By “high-fidelity transcript” we mean a verbatim, time-stamped, speaker-attributed record whose word error rate (WER), speaker-attribution accuracy, and clinical-concept fidelity are measured and reported. A transcript captures what was said and, through it, the reasoning that was explicitly communicated; further inferences from the transcript are possible but weaker; and the clinician's full internal reasoning (drawing on prior records, examination and imaging findings, pattern recognition, and tacit knowledge) remains only partly accessible. Our claim throughout is therefore about communicated reasoning: ambient transcripts make parts of communicated clinical reasoning observable and auditable at scale; they do not make clinical reasoning itself fully visible. Finally, we use “clinical audit” for the structured comparison of recorded practice against explicit, predefined criteria, “audit and feedback” for the return of audit results to clinicians, and “quality improvement” for the broader set of activities into which both feed; the terms are related but not interchangeable.

Each contribution illuminates one face of the same object: what is legally permitted, what is commercially practiced, and what is coming. Yet none directly addresses the question that determines all the others: what is the recording for?

The answer is not, in the first instance, legal. It has been articulated for nearly two decades, and reaffirmed in 2021, in the WHO work on audit and feedback to improve health care quality and safety (4). It also appears in the WHO Global Patient Safety Action Plan 2021–2030, which calls for the systematic analysis of clinical care data to generate learning and eliminate avoidable harm (5). For years, audit at scale remained out of reach, because the material needed to reconstruct clinical reasoning was simply unavailable. Recording the dialogue between clinicians and patients, together with a high-fidelity transcript, finally makes the communicated part of this reasoning visible.

Documentation, workflow efficiency, patient recall, medico-legal transparency, and quality improvement are all legitimate purposes of consultation recording, and they can coexist. Our claim is deliberately narrower: among these, one high-value governance purpose, clinical audit, has been under-recognized, and policy should make room for it rather than allowing presumed liability alone to define the field. Table 1 summarizes the stakeholder perspectives examined below.

**Table 1:** Stakeholder perspectives on transcript-based clinical audit: primary interests, principal concerns, and audit-ready requirements.

Stakeholder	Primary interest	Principal concern	What the audit-ready standard requires
Regulators	Lawful, safe deployment with a clear intended use	Privacy risks; high-risk AI obligations under the GDPR and the AI Act	Documented clinical purpose; compliance with data-protection and high-risk obligations; post-market monitoring
Vendors	Adoption driven by documentation efficiency	Cost and liability of audit-grade features	Timestamped speech turns; speaker diarization; word error rates reported and disaggregated by language and population
Patients	Safe, high-value care; being heard and protected	Intrusion, coercion, secondary use of data	Genuinely free consent with no consequence for declining; audio deletion after a short, disclosed verification window; pseudonymized transcripts
Vulnerable and under-represented populations (minors, cognitive impairment, low health literacy, interpreter-mediated care, psychiatric and other highly sensitive care)	Equitable protection; consent they can meaningfully exercise	Consent processes that are not intelligible to them; lower transcription accuracy; heightened sensitivity of content	Adapted consent materials and interpreter support; recording default-off where valid consent is impracticable; WER reported for their languages and contexts; heightened confidentiality safeguards
Clinicians	Reduced documentation burden; fair professional evaluation	Surveillance, discipline, defensive practice	Firewall between formative audit and discipline; right to review and contest findings; versioned guideline corpus
Health systems	Efficiency gains converted into quality improvement	Implementation cost, workforce, and governance burden	Independent audit body; stratified case selection; auditor training and inter-rater reliability checks
Auditors/quality bodies	Reliable, contestable measurement of care quality	Transcript fidelity; retroactive judgment against later standards	Explicit predefined criteria; second-reviewer adjudication of disagreement; documented appeal mechanism

## 2 The European regulatory perspective

European law allows this purpose but requires proper safeguards. Under the GDPR, consultation audio is personal data and, because it captures what patients disclose about their condition, will ordinarily constitute special-category data concerning health under Article 9 (6). Voice recordings additionally qualify as biometric data only where they undergo specific technical processing intended to uniquely identify a natural person, such as matching against a voiceprint; routine transcription, and diarization that merely distinguishes speakers within a single encounter, do not by themselves meet that definition (6). Under the EU AI Act, systems used to monitor and evaluate the performance and behaviour of persons in work-related relationships fall within the high-risk categories of Annex III; an audit tool deployed by a hospital or employer to evaluate clinicians would therefore likely be classified as high-risk, triggering obligations of risk management, data governance, transparency, human oversight, and post-market monitoring (7). Formal classification must nonetheless be assessed case by case, in light of the system's intended purpose and actual deployment context. These texts require reliability, transparency, and human oversight. They say how to deploy them correctly, not why. By “permits this purpose but does not supply it” we mean exactly this asymmetry: nothing in either text prohibits transcript-based audit conducted on a valid legal basis with appropriate safeguards, yet neither text articulates the clinical objective that would justify it. That objective must come from health policy. This regulatory grey zone (binding rules on deployment without an articulated clinical purpose) has been characterized for clinical AI more broadly (8).

The WHO Global Patient Safety Action Plan 2021–2030 explicitly identifies digital technology, including artificial intelligence, as a strategic enabler for safer health care (Strategy 6.5) (5). When regulation lacks an explicit clinical purpose, it tends to remain defensive. The WHO framework supplies that purpose, and privacy by design becomes a means rather than an end.

Prompt audio deletion minimizes privacy risk, but immediate deletion also forecloses later verification of transcript accuracy, a genuine trade-off. A proportionate design is a short, access-controlled audio-retention window, for instance until the clinician has validated the transcript, or a fixed brief period reserved for quality-assurance sampling, after which the audio is irreversibly deleted. Pseudonymized transcripts then enable quality audits against current guidelines. European law supports responsible deployment. The same clinical-audit purpose could extend to low- and middle-income settings. There, as the WHO Global Patient Safety Report 2024 documents, the burden of avoidable harm is greater, and safety in primary and ambulatory care remains the least prioritized (9). A transcript-based approach should therefore be designed for these settings from the outset, not retrofitted after high-income adoption.

## 3 The vendor perspective

Vendors market ambient AI as a documentation and administrative tool, which is understandable amid clinician burnout. However, this framing limits the technology's scope and clinical value. Reasoning that is never recorded can be audited only indirectly, through notes, coded data, outcomes, peer review, and case discussion. These channels remain essential, but none captures diagnostic thinking, the alternatives weighed, or the patient's expressed preferences as directly, or at comparable scale, as a faithful transcript. Auditing transcripts, not merely transcribing them, is what makes this reasoning verifiable against guidelines. Even then, clinically important reasoning is not always fully verbalized, so transcript-based audit is powerful but not exhaustive.

Empirically, vendor practices reflect this narrow focus. Gerke and Simon document that, at present, there is no harmonized standard governing how commercial scribes handle audio, transcripts, and clinical notes (2). To meet the WHO call for clinical care audits to feed national patient safety information systems (Strategy 6.2) (5), vendors must redesign their products to be audit-ready. This requires more than note summarization. It demands systematic timestamping of speech turns, speaker diarization (clinician, patient, companion), and transparent reporting of each system's WER. Crucially, WER is not uniform across speakers. Recognition trained largely on high-resource languages and dominant accents performs less well for other languages, dialects, and populations (10). Unequal transcription accuracy then risks unequal audit reliability, and unequal protection for the patients least well served. WHO guidance on the ethics and governance of AI for health makes inclusiveness and equity a core requirement, and cautions that systems built mainly on high-income-country data may underperform in low- and middle-income settings (11). Developers should accordingly report WER disaggregated by language and population rather than as a single aggregate figure. Audit software providers, in turn, must guarantee transparency, security, and reliability, and must benchmark transcripts against contemporaneous guidelines. Auditing a past encounter against an updated guideline would unfairly judge, retroactively, a decision that was appropriate when it was made.

Our pilot study, currently available as a preprint that has not yet undergone peer review (12), suggests the feasibility of a transcript-based audit-and-feedback loop in everyday clinical practice, and its conclusions should be read with corresponding caution. Challenges remain, however. They include variable transcription accuracy in complex consultations, the need for rigorous human oversight, and practical integration at scale. This approach differs from real-time decision support, whose repeated use may reduce cognitive availability during the encounter and create a risk of automation bias (13), although direct comparative evidence remains limited. Audit of the transcript, by contrast, examines reasoning after the fact. It preserves the clinician's attention while producing a verifiable record.

## 4 The patient perspective

Under the GDPR, explicit consent will often be required, or will be the most appropriate lawful basis, for consultation recording in the use we propose; the precise lawful basis must nonetheless be determined under applicable national law and institutional arrangements, and consent to recording remains distinct both from consent to care and from the lawful basis for any subsequent processing (6). Yet current institutional frameworks present this consent mainly as a service to the institution. This framing disadvantages patients, who accept the risk of an intrusion into their privacy without any clear, direct clinical benefit.

Consent should be reframed around patient protection. Recording preserves the integrity of the exchange and enables continuous eye contact between patient and clinician. It produces a record that external auditors can compare with practice guidelines. Where an estimated 20% of care is deemed unnecessary or low value, a figure that varies substantially across settings and definitions (14), the transcript could serve as an accountability tool aligned with the WHO ambition (4). Was the indication justified? Were non-invasive options discussed honestly and presented? The transcript can help answer these questions more transparently. Patients should consent not for institutional convenience, but to be heard and protected by audits that support the clinical value of their care.

Consent must remain genuinely free, and its protective framing is a hypothesis to be tested rather than assumed. Some patients will experience recording as intrusive, coercive, or corrosive of trust, particularly in psychiatric and other sensitive consultations, and declining must carry no consequence for access to care, for the clinician's attention, or for the quality of documentation. Specific safeguards are needed for minors, for patients with low literacy, and for multilingual encounters, where the information supporting consent must itself be intelligible; in emergencies, where valid consent is impracticable, recording should default to off.

## 5 The clinician perspective

Consent built around audit also protects clinicians. When a clinician seeks explicit consent for recording and presents it as a commitment to quality assurance, they do not hide behind technology but make the consultation open to review. A transcript that clearly shows reasoning consistent with the guidelines can be a valuable artifact in litigation or professional review, demonstrating alignment between what was said, what was heard, and what was recommended. It is not inherently protective in every dispute, and its value depends on transcription accuracy and context. This approach must still address clinicians’ legitimate concerns about perceived surveillance or added burden, and a further risk deserves naming: fear of retrospective scrutiny could encourage defensive, performative verbalization rather than better care. Parallel risks arise on the audit side: reviewers presented with automated flags may exhibit confirmation bias, anchor on a machine-generated interpretation, over-rely on algorithmic assessment, or progressively deskill; on the clinical side, awareness of recording may alter clinician–patient communication, and speech or documentation may drift toward satisfying anticipated audit criteria rather than serving the patient. How recording and auditing change clinician behaviour, and the authenticity of the consultation itself, should be studied as outcomes in their own right rather than assumed to be neutral. The medico-legal discoverability of transcripts, moreover, varies by jurisdiction and requires explicit local clarification before deployment. Only transparent governance and professional participation can mitigate these concerns.

## 6 Implementation governance

Operationalizing transcript-based audit requires explicit governance. Audits should be conducted by a body independent of line management, such as a quality-and-safety committee or a peer-review panel, rather than by an employer acting as both auditor and judge. Where full structural independence is not feasible, as in smaller institutions and resource-constrained settings, governance should at minimum ensure functional separation, transparent membership, declared conflicts of interest, multidisciplinary representation, documented procedures, and meaningful rights of review and appeal. Cases should be selected by random sampling stratified by clinical risk, complemented by triggered review of predefined sentinel situations, so that routine encounters are represented while high-impact decisions receive systematic attention. Each audit must apply explicit, predefined criteria, anchored to the specific guideline version in force on the date of the consultation, through a versioned and time-stamped reference corpus, so that no decision is judged against a standard published later. Where reviewers disagree, a second independent reviewer should be engaged, with formal adjudication of the disagreement. Clinicians should have a documented right to review and contest any finding before it is recorded, with a defined appeal mechanism. Auditor training and periodic inter-rater reliability checks are equally important, since the credibility of any finding rests on consistent, calibrated human judgment. Finally, governance must distinguish guideline non-conformity from substandard care: a justified, documented departure from a guideline in the patient's interest is not an error, and audit should surface the reasoning rather than enforce mechanical compliance.

The audit instrument itself requires formal development and validation before deployment: explicit operational definitions, structured reviewer guidance, pilot testing, assessment of content validity, calibration exercises, and monitored inter-rater and intra-rater reliability, together with defined procedures for missing or ambiguous information and periodic review of the framework as guidelines and practice evolve. Uncertainty or incomplete verbalization must not default to a finding of non-conformity: the instrument should provide an explicit “indeterminate” or “insufficient information” outcome rather than forcing a binary compliant/non-compliant judgment.

Transcript fidelity must likewise be distinguished from the validity of the conclusions drawn from it: an accurate transcript can still be misread, by an automated system or a human auditor, when a decision rested on contextual, longitudinal, or unspoken information. Findings should therefore normally be interpreted alongside the relevant clinical record (medication history, test results, prior consultations, physical findings, and subsequent events) rather than in isolation, and future validation studies should assess two levels separately: the fidelity of the transcript to the original encounter, and the validity, reproducibility, and clinical relevance of the audit conclusions derived from it, benchmarked against independent expert review of the complete clinical record, with attention to agreement measures, false-positive and false-negative findings, and the consequences of disagreement.

Formative audit and feedback, professional peer review, quality improvement, disciplinary investigation, and medico-legal evidence are different activities governed by different rules; data collected for formative purposes should be firewalled from performance management and discipline, and any crossing of that line should require due process and be stated explicitly in consent materials. Without this separation, transcript-based audit risks sliding into workplace surveillance, precisely the outcome that would forfeit clinician trust and, with it, the patient-safety purpose.

These requirements converge with emerging frameworks for algorithmovigilance and post-deployment governance of clinical AI, which emphasize intended use, continuous monitoring, human oversight, and organizational accountability after implementation (8, 15).

## 7 The efficiency dividend: documented benefits and time saved

Reframing recording around audit does not require abandoning the efficiency case; it absorbs it. The documentation burden that ambient AI addresses is a worldwide problem. Across health systems, clinicians spend a substantial share of their working time on electronic documentation rather than on patients, a recognized driver of burnout. A systematic review of ambient AI scribes across multiple countries found consistent reductions in documentation burden and cognitive load, improved workflow efficiency, and better clinician-patient interaction. It stressed, however, that the evidence remains heterogeneous and that clinician oversight of AI-generated notes is essential (16). Beyond self-report, a prospective time-motion study in Singapore used direct observation of routine outpatient consultations to measure documentation time, consultation duration, and clinician-patient eye contact, providing objective corroboration in a non-Western setting (17).

These benefits are real but uneven. Their magnitude depends on usage intensity, specialty, language, and implementation. Syntheses also caution that some studies show only modest time savings, and that AI-drafted notes can contain errors or omissions requiring clinician review (16). Benefits should therefore be measured locally rather than assumed and, consistent with WHO guidance on AI for health, must not be allowed to accrue mainly to well-resourced, dominant-language settings (11). Critically, the efficiency dividend and the audit purpose are not competing goals; they draw on the same artifact, the recorded and transcribed encounter. Time savings will drive adoption, while clinical audit could convert that adoption into value for patients, a conversion that is itself a hypothesis requiring prospective evaluation. To frame ambient AI solely as a time-saver would repeat the very narrowing this paper warns against.

## 8 Discussion

The policy case for audit and feedback is now compelling. Several years of research and evidence show that audit and feedback can meaningfully improve professional practice and patient outcomes (18). What was historically missing was not the rationale but a scalable way to reconstruct the communicated part of clinical reasoning, which ambient AI now supplies. Whereas written clinical reports summarize outcomes and lack the nuance of physician reasoning, ambient AI makes that reasoning available. The proposal sits within the broader trajectory of patient-safety research and clinical risk management, which has moved from incident reporting toward continuous, technology-enabled learning systems, and it inherits that field's central lesson: measurement improves care only when embedded in just and transparent governance.

The policy conditions for action are now in place. An exclusively defensive reading of privacy and legal risk may inadvertently delay safety-oriented learning systems and the patient protection they offer (14). Practical and organizational challenges remain: audit scalability, transcription reliability, and team buy-in. But these are challenges of implementation, not of principle. This reframing also addresses a concern recently voiced by Kohane (19), that AI applied to clinical guidelines could end up generating and enforcing recommendations at scale, at the expense of clinical judgment. Transcript-based audit inverts that logic. Retrospective, pedagogical, and contestable by design, it targets errors of omission rather than enforcing compliance, thereby preserving the discretionary space in which good medicine occurs (19). Because clinical reasoning is partly tacit, contextual, and shaped by nonverbal interaction, transcript-based audit should complement, not replace, other quality signals such as outcomes, peer review, and direct observation.

This Perspective has limitations. It is a policy argument, not an empirical evaluation, and the framework it proposes has not been prospectively validated; the pilot study we cite is currently a preprint (12). The feasibility and cost of independent audit at scale remain uncertain, and legal bases, discoverability, consent requirements, and professional protections vary across jurisdictions. Transcript-based audit cannot capture all clinically relevant information, and its effects on patient trust and clinician behaviour are unknown. The minimum standard we propose will likely require substantial adaptation across specialties, languages, health systems, and resource settings. Our conclusions should therefore be read as a proposal for testing and implementation research, not as an established model of patient-safety improvement.

Audited transcripts turn the consultation into a source of learning and accountability, directed toward a single end: the clinical value of the care a patient receives.

## 9 Conclusion: a key action

Ambient AI developers, regulators, professional bodies, and health systems should adopt a minimum audit-ready transcript standard as a precondition for procurement, aligned with the WHO Global Patient Safety Action Plan 2021–2030 (5) and WHO guidance on AI for health (11): timestamped speech turns, speaker diarization, audio deletion after a short, disclosed verification window, pseudonymized transcript retention, and WER reported and disaggregated by language and population. Prospective validation across specialties, languages, and care settings will be needed before such a standard could justify binding procurement requirements. To advance global equity, this standard should be piloted in parallel across high-income and low- and middle-income settings, so that the clinical value of ambient AI is built in from the outset rather than retrofitted after high-income adoption. Parallel piloting will not happen by aspiration alone. It requires speech models and word-error-rate benchmarks for local languages, drawing on open-source models where commercial coverage is absent; investment in data-protection capacity; deployment modes tolerant of limited connectivity; an audit workforce trained within existing quality structures; and procurement frameworks, potentially coordinated through WHO and regional bodies, that make the standard a condition of purchase rather than an afterthought.

Pilots should predefine their outcome measures: transcription and speaker-attribution accuracy; clinical-concept fidelity; agreement between transcript-based audit and independent expert review; the frequency of indeterminate judgments; clinician and patient acceptability; effects on trust and communication; the time and cost of auditing; equity of performance across languages and populations; rates of contested findings; changes in professional practice; unintended consequences; and patient-safety outcomes. An audit-ready transcript standard is, finally, deliberately narrower than a full audit-system standard: it specifies the technical properties of the record, while the governance described in Section 6 specifies how audits based on that record must be conducted; both are needed, and neither substitutes for the other.

## Data Availability

The original contributions presented in the study are included in the article/Supplementary Material, further inquiries can be directed to the corresponding author.
